# Targeting cellular senescence to combat cancer and ageing

**DOI:** 10.1002/1878-0261.13266

**Published:** 2022-06-20

**Authors:** Chen Wang, Xue Hao, Rugang Zhang

**Affiliations:** ^1^ Immunology, Microenvironment & Metastasis Program The Wistar Institute Philadelphia PA USA

**Keywords:** ageing, cancer, senescence, senolytics, senomorphics

## Abstract

Senescence is a complex cellular process that is implicated in various physiological and pathological processes. It is characterized by a stable state of cell growth arrest and by a secretome of diverse pro‐inflammatory factors, chemokines and growth factors. In this review, we summarize the context‐dependent role of cellular senescence in ageing and in age‐related diseases, such as cancer. We discuss current approaches to targeting senescence to develop therapeutic strategies to combat cancer and to promote healthy ageing, and we outline our vision for future research directions for senescence‐based interventions in these fields.

## Graphical abstract

Targeting senescence to treat cancer and ageing raises increasing enthusiasm in both academia and industry. Current approaches to target senescence with a discussion of their benefits and drawbacks have been proposed. This review also explores future research directions for senescence‐based interventions to combat cancer and ageing.

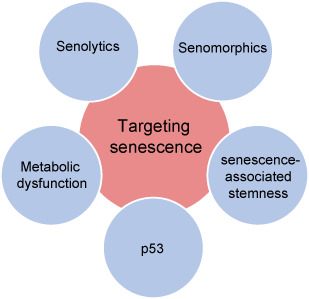

**Abbreviations**
2,3‐cGAMP: 2,3‐cyclic GMP‐AMP
AP‐1: activator protein 1
BET: Bromodomain and extra‐terminal domain
BRD4: Bromodomain containing 4
C/EBPβ: CCAAT/enhancer‐binding protein b
CAR: chimeric antigen receptor
CCF: cytoplasmic chromatin fragments
cGAS: cyclic GMP‐AMP synthase
DDR: DNA damage response
DOT1L: telomeric silencing 1‐like
eccDNAs: extrachromosomal circular DNA elements
EGF: endothelial growth factor
GATA4: GATA‐binding protein 4
GLS1: glutaminase 1
H3K4: histone H3 lysine 4
HFD: human fetal diploid
HMGA: high mobility group A
HUVECs: human umbilical vein endothelial cells
ICB: immune checkpoint blockade
IL1A: interleukin 1 Alpha
IRFs: interferon regulatory transcription factors
JAK2: Janus Kinase 2
JMJD3: Jumonji domain containing 3
JNK: c‐Jun N‐terminal kinase
KDM4: histone lysine demethylase subfamily 4
LINE1: long‐interspersed element 1
LSECs: liver sinusoidal endothelial cells
m^6^A: N6‐methyladenosine
MAPK: mitogen‐activated protein kinase
METTL3: methyltransferase‐like 3
MiDAS: mitochondrial dysfunction‐associated senescence
MLL1: mixed lineage leukaemia protein‐1
mtDNA: mitochondrial DNA
NAMPT: nicotinamide phosphoribosyl transferase
NFκB: nuclear factor‐kappa B
NGS: next‐generation sequencing
NHEJ: non‐homologous end joining
OIS: oncogene‐induced senescence
PARP: poly‐ADP‐ribose polymerase
PD‐1: programmed cell death protein 1
ROS: reactive oxygen species
RS: replicative senescence
SAHF: senescence‐associated heterochromatic foci
SASP: senescence‐associated secretory phenotype
SCAPs: senescent‐cell anti‐apoptotic pathways
scRNA‐sequencing: single‐cell RNA sequencing
scSPRITE: single‐cell split‐pool recognition of interactions by tag extension
SIPS: stress‐induced premature senescence
SIRTs: sirtuin family proteins
SOD2: mitochondrial superoxide dismutase
STING: stimulator of interferon genes
TGFβ1: transforming growth factor beta 1
TIS: therapy‐induced senescence
TOP1: topoisomerase 1
uPAR: urokinase‐type plasminogen activator receptor
VEGF: vascular endothelial growth factor

## 1 Introduction

In response to cellular stressors, cells undergo senescence, a cellular state that is characterized by the stable arrest of cell growth. While in the senescent state, cells secrete a variety of pro‐inflammatory factors, chemokines and growth factors, which together are known as the senescence‐associated secretory phenotype (SASP) [1, 2]. Senescent cells also undergo a significant amount of chromatin remodelling, DNA damage response (DDR), specific morphological changes, and enter into an altered metabolic state [1].

**Fig. 1:**
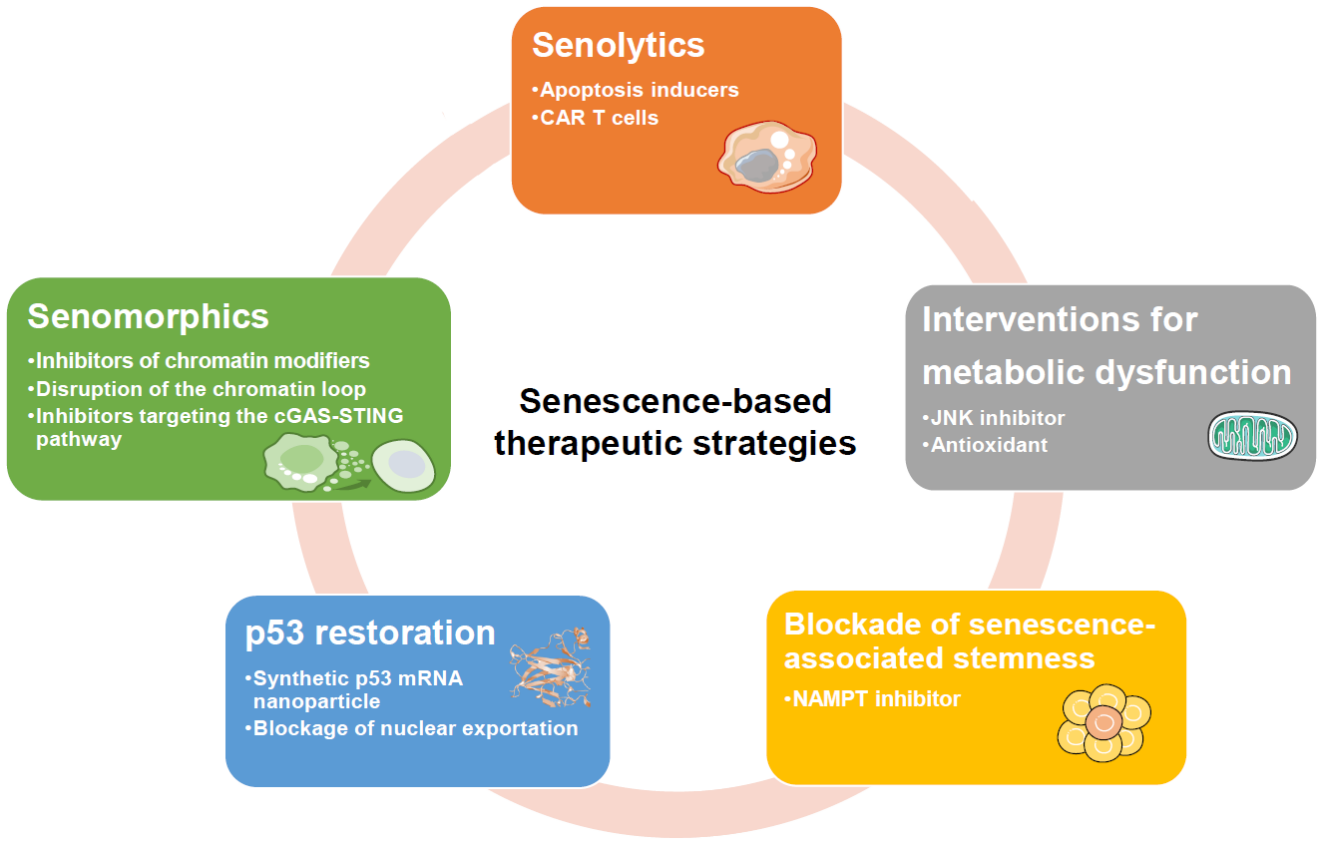
The potential of senescence‐based therapeutic strategies for promoting healthy ageing and for combating age‐related disease. These strategies include the development of Senolytics and Senomorphics, interventions for metabolic dysfunction, p53 restoration, and the blockade of senescence‐associated cancer cell stemness. CAR, chimeric antigen receptor; cGAS, cyclic GMP‐AMP synthase; JNK, c‐Jun N‐terminal kinase; NAMPT, nicotinamide phosphoribosyl transferase; STING, stimulator of interferon genes. [Colour figure can be viewed at wileyonlinelibrary.com]

Senescence can be highly variable and heterogenous. There are four main types of senescence that cells can undergo, depending on the inducer: replicative senescence (RS); oncogene‐induced senescence (OIS); stress‐induced premature senescence (SIPS) and therapy‐induced senescence (TIS) [3, 4, 5, 6, 7]. These diverse types of senescence are implicated in a variety of physiological and pathological processes, and modulate the tissue microenvironment mainly through the SASP [8]. Senescence and the SASP are also widely reported to have a context‐dependent role in tissue ageing and in age‐associated diseases, such as cancer [9]. Indeed, senescence‐based interventions for ageing and age‐associated diseases have gained substantial traction with an increasing number of papers being published each year [10, 11, 12, 13]. This review explains the rationale of targeting senescence as an effective strategy to combat age‐related disorders with a particular focus on cancer. We discuss both the benefits and challenges of current senescence‐targeting approaches and outline our vision for future senescence‐based intervention strategies in promoting healthy ageing and the treatment of cancer and other age‐related diseases (Fig. 1).

## 2 Clearance of senescent cells by senolytics

Senescence plays a context‐dependent role in cancer and in other diseases, such as lung fibrosis [14, 15]. In response to versatile stress inducers, senescent cells can be either beneficial or detrimental to tissues through remodelling the tissue microenvironment [1]. The detrimental effect induced by senescent cells addresses ideas on developing ‘senolytics’, which aim at eliminating senescent cells from damaged tissues [16]. This therapeutic strategy is expected to restore tissue homeostasis, reduce age‐associated pathology, and treat age‐related diseases.

### 2.1 Cellular senescence in cancer and ageing

**Fig. 2:**
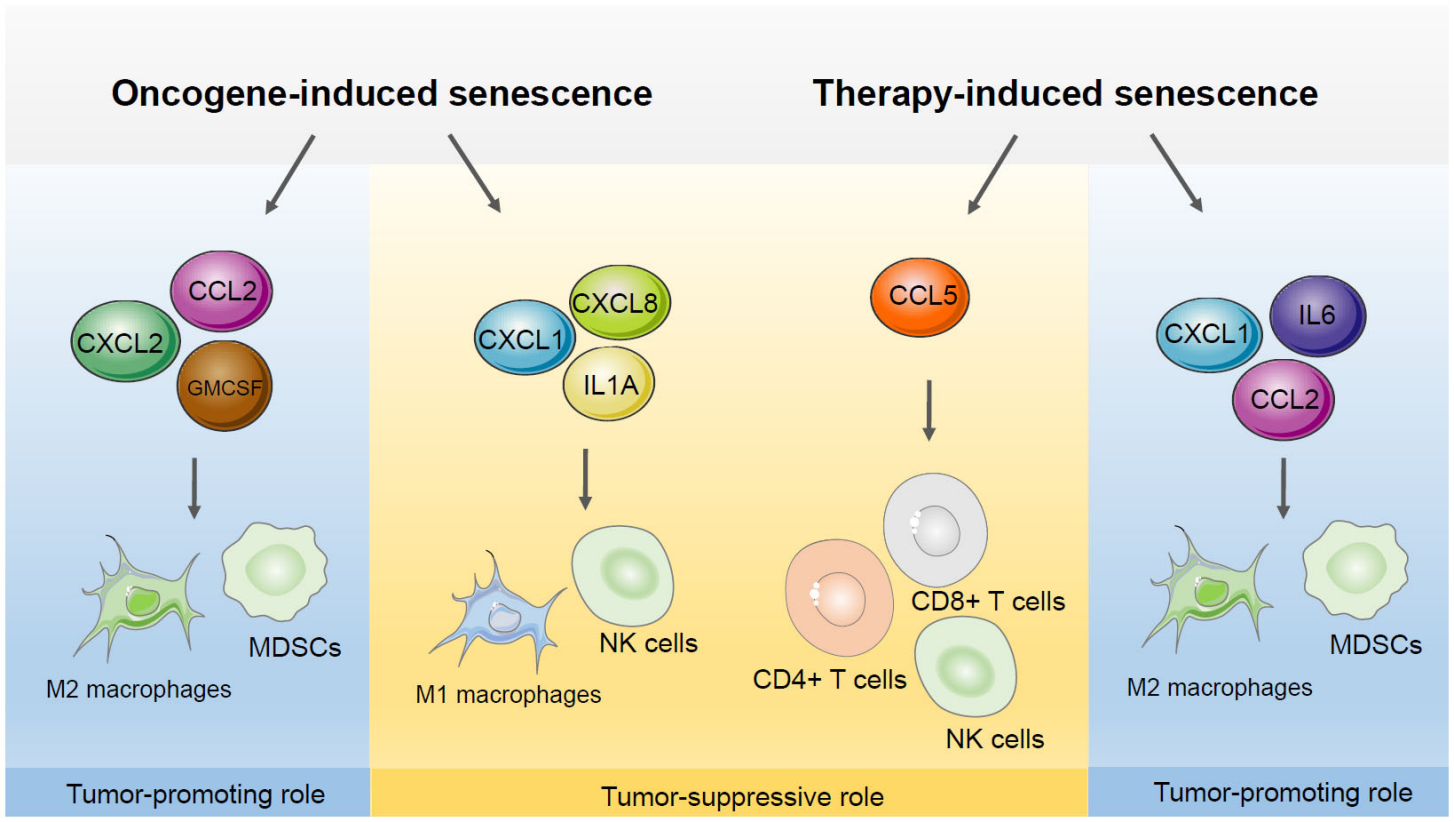
The multifaceted roles of the SASP in different context of senescence. The tumour‐promoting (blue) and the tumour‐suppressive (orange) roles of the SASP are summarized. In different senescence settings, the SASP factors can be highly variable and result in different biological functions. For example, CXCL1 secreted by OIS cells is important in suppressing tumour progression, while promoting tumour growth during TIS. CCL2, C‐C Motif Chemokine Ligand 2; CCL5, C‐C Motif Chemokine Ligand 5; CD4, Cluster of Differentiation 4; CD8, Cluster of Differentiation 8; CXCL1, Chemokine (C‐X‐C motif) ligand 1; CXCL2, Chemokine (C‐X‐C motif) ligand 2; CXCL8, Chemokine (C‐X‐C motif) ligand 8; GMCSF, Granulocyte‐macrophage colony‐stimulating factor; IL1A, Interleukin 1 Alpha; IL6, Interleukin 6; MDSCs, Myeloid‐derived suppressor cell; NK, natural killer; OIS, oncogene‐induced senescence; SASP, Senescence‐associated secretory phenotype; TIS, therapy‐induced senescence. [Colour figure can be viewed at wileyonlinelibrary.com]

OIS is an intrinsic tumour suppression mechanism. It prevents premalignant cells that harbour an initial oncogenic hit from becoming fully transformed cancer cells [17]. However, OIS has also been shown to contribute to tumour development. It does so by remodelling the tumour immune microenvironment through the SASP, which is characterized by the secretion of pro‐inflammatory factors, chemokines and growth factors (Fig. 2). For example, CCL2 secreted by cells undergoing OIS promotes cancer progression by recruiting immune‐suppressive M2 polarized macrophages [18]. Likewise, docetaxel‐induced senescence can reduce the size of *Pten*‐deficient tumours by triggering immunosurveillance when combined with Janus Kinase 2 (JAK2) inhibitor NVP‐BSK805 [19]. However, persistent TIS increases the risk of cancer relapse and chemoresistance by inducing cancer stem‐like cells [20, 21, 22]. In addition, TIS contributes to chemotherapy‐induced side effects by stimulating persistent local and systemic inflammation [20].

Notably, the elimination of senescent cells delays tumorigenesis [23]. The clearance of senescent cells by immune cells has also been reported to reduce fibrosis in multiple organs and maintain uterine function in wild‐type mice [24, 25]. Therefore, the selective removal of senescent cells is considered to be a potential therapeutic strategy with which to treat cancer and promote healthy ageing.

### 2.2 Using senolytics to eliminate senescent cells

Caloric restriction in wild‐type mice is known to prolong a healthy lifespan by delaying the accumulation of senescent cells [26, 27]. This finding links senescent cell burden to ageing [27]. Subsequent work in mice and in human pre‐malignant tumours has established that senescent cells play a causal role in driving ageing and age‐related diseases, including cancer [12, 28]. The elimination of senescent cells from damaged tissues in wild‐type mice relieves the symptoms of senescence‐related disorders, restores tissue homeostasis, and promotes longevity [29]. Consequently, increasing efforts have been devoted to developing ‘senolytics’ that can selectively eliminate senescent cells.

The first generation of senolytics was discovered based on the intrinsic resistance of senescent cells to apoptosis [30]. Senescence in cells is accompanied by the upregulation of senescent‐cell anti‐apoptotic pathways (SCAPs), which reinforce the senescence status in cells, preventing them from undergoing apoptosis [31, 32]. One such pathway is the BCL‐2 pro‐survival pathway, members of which include ABT‐737 (Table 1). Pan inhibitors of BCL‐2 pathway members trigger the apoptosis in OIS and TIS and in normal‐aged mice [33, 34]. In wild‐type aged mice treated with a combination of apoptosis activators, lifespan was extended, even when this treatment was administered in later life [35]. However, the non‐specificity of these compounds may cause toxic side effects, which prohibit their use in a therapeutic context [36]. A specific BCL‐xL inhibitor ABT‐263 (navitoclax) that is less hematologically toxic than the pan inhibitors was shown to selectively induce apoptosis in senescent human umbilical vein endothelial cells (HUVECs) but not in proliferating ones [37]. These studies establish the potential for repurposing apoptosis inducers as senolytics.

**Table 1:** Senolytics and senomorphics.

	Targets	Refs
Senolytics		
ABT‐263 (navitoclax)	BCL‐XL	[33, 37]
ABT‐737	BCL‐2, BCL‐XL and BCL‐W	[34]
Quercetin	Multiple targets	[35]
Dasatinib	Pan‐receptor tyrosine kinases	[36]
Fisetin	PI3K/AKT/mTOR	[38]
BET family protein degrader (ARV825)	Bromodomain and extraterminal domain family protein	[40]
BPTES	GLS1	[41]
uPAR‐specific CAR T cells	uPAR positive senescent cells	[29]
Senomorphics		
Metformin	IKK and/or NF‐κB	[67]
MI‐2‐2	MLL1	[69]
JQ1	BRD4	[55]
EPZ5676	DOT1L	[57]
ML324	KDM4	[56]
TSA	HDAC	[82]
SP600125	JNK	[101]
FK866	NAMPT	[22]

Another study has shown that flavonoid polyphenol fisetin can serve as a potent senolytic drug. This drug has been shown to reduce the senescent cell burden in wild‐type mice and in human adipose tissue explants [38]. More importantly, the administration of fisetin to wild‐type mice late in life significantly extended their median and maximum lifespan, without causing severe side effects. Given the safety profile of fisetin in humans, its efficacy in reducing markers of cellular senescence in elderly subjects is currently being assessed in a clinical trial [39]. Bromodomain and extra‐terminal domain (BET) family degrader also serve as potent senolytic drugs [40]. For example, the BET degrader ARV825 provokes senolysis through the combined effects of the attenuation of non‐homologous end joining (NHEJ) repair and the activation of autophagic pathway in RS cells [40]. Furthermore, as the intracellular pH during senescence is lowered which in turn stimulates glutaminase 1 (GLS1) expression, GLS1 inhibitor BPTES has been shown to eliminate senescent cells induced by transient p53 activation and ameliorate age‐associated organ dysfunction in aged mice [41]. In addition, a tolerated FOXO4‐p53 interfering peptide has been reported to promote nuclear exclusion of active p53 in infrared radiation (IR)‐induced senescent cells, which in turn induces p53‐mediated intrinsic apoptosis and restores health span in both fast‐ageing *Xpd* ^TTD/TTD^ and naturally aged mice [31].

In a recent study, urokinase‐type plasminogen activator receptor (uPAR) was identified as a prevalent cell‐surface and secreted senescence biomarker [29]. Although uPAR plays a role in cell motility and in tumour cell survival, mice lacking uPAR do not have altered fertility or development [42], which exhibits a promising safety profile. This suggests that the inhibition of uPAR could be potentially used in a therapeutic context. In a mouse model of lung adenocarcinoma, in which cell senescence is stimulated, the injection of a uPAR‐specific chimeric antigen receptor (CAR) T cells efficiently eliminated senescent cells and significantly extended survival [29]. CAR T cells are T cells that have been genetically engineered to produce a chimeric T‐cell receptor for use in immunotherapy. This study highlights the potential to treat age‐related diseases by senolytic CAR T cells.

As discussed above, several studies have demonstrated the beneficial effects of senolytics in the context of ageing and age‐associated disorders in animal models. However, whether they are equally effective in human clinical trials is yet to be determined given the paradoxical and context‐dependent role of senescence. Particularly, senolytics typically only eliminate a fraction of senescent cells and are known to induce side effects such as pleural effusion and dose‐limiting thrombocytopenia [16]. When treated with senolytics at effective doses, damaged osteoblast function and reduced trabecular bone volume fraction have been shown in wild‐type mice [43]. In addition, a recent study found that p16^High^ senescent cells are structurally and functionally important in ageing organisms [44]. Elimination of p16^High^ senescent liver sinusoidal endothelial cells (LSECs) could lead to disruption of blood‐tissue barriers and subsequent fibrosis [44]. Thus, caution should be taken when applying these drugs in a therapeutic context.

Given that the potential side effects caused by senolytics and that the SASP plays an important role in promoting ageing‐related diseases, targeting the SASP by senomorphics becomes an alternative option.

## 3 Targeting the SASP with senomorphics

The SASP plays a context‐dependent role in cancer and ageing [45, 46], and functions in a dynamic, variable, and heterogeneous manner in different senescence contexts [47]. Given that the detrimental effect caused by senescent cells largely depends on the SASP, senomorphics that aim at suppressing the SASP without affecting the senescence‐associated growth arrest attract broad academic interests.

### 3.1 Paradoxical role of the SASP in cancer and ageing

The SASP‐associated factors secreted from OIS cells can trigger immune surveillance to remove premalignant cells that harbour initial oncogenic hits [46]. This immune surveillance, together with senescence‐associated growth arrest, enforces tumour suppression [48]. Consistent with its role in boosting the immune response, the SASP benefits cancer immunotherapies by improving the efficacy of the immune checkpoint blockade (ICB), such as by sensitizing programmed cell death protein 1 (PD‐1) inhibitors [49]. PD‐1 inhibitors block the activity of PD‐1 immune checkpoint proteins present on immune cells, which are involved in the suppression of immune response [50]. Likewise, CDK4/6 inhibitors have been shown to induce the SASP and to recruit CD8 T cells to overcome resistance to ICB [51, 52]. Additionally, topoisomerase 1 (TOP1) functions to relax high‐order topological DNA structures during DNA replication and transcription [53]. TOP1 inhibitors sensitize ovarian cancers to ICB by enhancing the expression of SASP‐associated factors [54]. However, the SASP can be detrimental to health as it is a source of chronic inflammation in many age‐related diseases, including in cancer [46]. For example, some SASP‐associated factors, such as vascular endothelial growth factor (VEGF), endothelial growth factor (EGF) and transforming Growth Factor Beta 1 (TGFβ1), promote tumorigenesis and tumour progression [45]. Given the multifaceted role of the SASP in different settings, it should be modulated with precision when being targeted for therapeutic purposes.

### 3.2 Using senomorphic drugs to suppress SASP

Given that eliminating senescent cells by senolytics can cause unwanted side effects and that the detrimental effects of senescent cells are largely mediated by the SASP, an alternative approach to targeting senescent cells is to develop senomorphics. Senomorphic drugs, such as BET inhibitor I‐BET‐762 and histone lysine demethylase subfamily 4 (KDM4) inhibitor ML324, aim to suppress the SASP [55, 56]. Notably, senomorphics are expected not to affect the arrest of cell growth that is associated with senescence [57].

#### 3.2.1 Suppressing SASP by targeting chromatin modifiers

OIS cells have long been known to induce the formation of visible senescence‐associated heterochromatic foci (SAHF) [58]. These foci form when already existing heterochromatin is repositioned by chromatin modifiers, such as Jumonji domain containing 3 (JMJD3) [59, 60]. The formation of SAHF functions as a safeguard as SAHF suppresses the transcription of proliferation‐promoting genes in OIS cells [59]. In addition, the transcription of genes that encode SASP‐associated factors correlates with the exclusion of their loci from SAHF, as evidenced by an accessible genomic locus adjacent to the SAHF in OIS cells [61]. Transcription factors also play key roles in regulating the SASP. For example, nuclear factor‐kappa B (NFκB), CCAAT/enhancer‐binding protein b (C/EBPβ) and Activator protein 1 (AP‐1) are transcription factors that regulate the transcription of genes encoding SASP‐associated factors in OIS cells [62, 63, 64, 65]. Additionally, GATA‐binding protein 4 (GATA4) indirectly induces the SASP by interacting with NFκB in OIS, RS, and IR exposed cells [66]. Moreover, an anti‐diabetic drug Metformin has been reported to reduce the SASP through inhibition of NFκB activation [67]. Finally, an RNAi screen for SASP regulators identified 50 targets such as PTBP1, which are druggable to suppress the inflammatory secretome [68].

Target SASP‐promoting transcription factors is challenging due to the fact that they lack catalytic active sites for drugs to bind. Therefore, chromatin modifiers that have drug binding pockets become ideal targets for suppressing the SASP. For example, mixed lineage leukemia protein‐1 (MLL1), which mediates histone H3 lysine 4 (H3K4) methylation, contributes to SASP upregulation in both OIS and TIS models [69]. Consistently, MLL1 inhibition reduces SASP gene expression via the DNA damage and response pathway. Additionally, disruptor of telomeric silencing 1‐like (DOT1L) is required for Interleukin 1 Alpha (IL1A) expression, which is an important upstream regulator of many other SASP genes [70]. Furthermore, inhibition of the epigenetic regulator Bromodomain Containing 4 (BRD4), which helps to organize new super enhancers to drive the SASP, suppresses this secretory phenotype [55]. Similarly, a potent inhibitor that selectively targets KDM4 can also reduce SASP gene expression by altering the accessibility of chromatin and the transcriptomic landscape [56]. Importantly, the inhibition of these chromatin modifiers reduces SASP gene expression without altering the growth arrest of senescent cells, which provides a promising avenue for being targeted in the therapeutic context.

In addition, a recent study has shown that the disruption of the chromatin loop that drives the expression of SASP‐associated genes might also be used to reduce the SASP [71]. The methyltransferase‐like 3 (METTL3) and 14 (METTL14) catalyze mRNA N^6^‐methyladenosine (m^6^A) modification [72]. However, in senescent cells, the METTL3 and METTL14 complex mediates important senescence‐associated enhancer‐promoter (EP) looping in an enzymatic activity‐independent manner [71]. Specifically, Mettl3 and Mettl14 redistribute to the promoter and enhancer of SASP‐associated genes, respectively [71]. In wild‐type mouse models, genetically knockdown of METTL3 or METTL14 reduces the immune surveillance function of the SASP. In addition, in a xenograft mouse model, knockdown of Mettl3, Mettl14 significantly decreases the tumorigenesis mediated by the SASP [71]. Notably, SASP reduction brought about by Mettl3 and Mettl14 depletion was not accompanied by impairments to the senescence‐associated growth arrest [71]. Thus, the Mettl3/Mettl14 complex could provide a promising target for new anti‐SASP senomorphics.

#### 3.2.2 Targeting cGAS‐STING pathway to suppress SASP

**Fig. 3:**
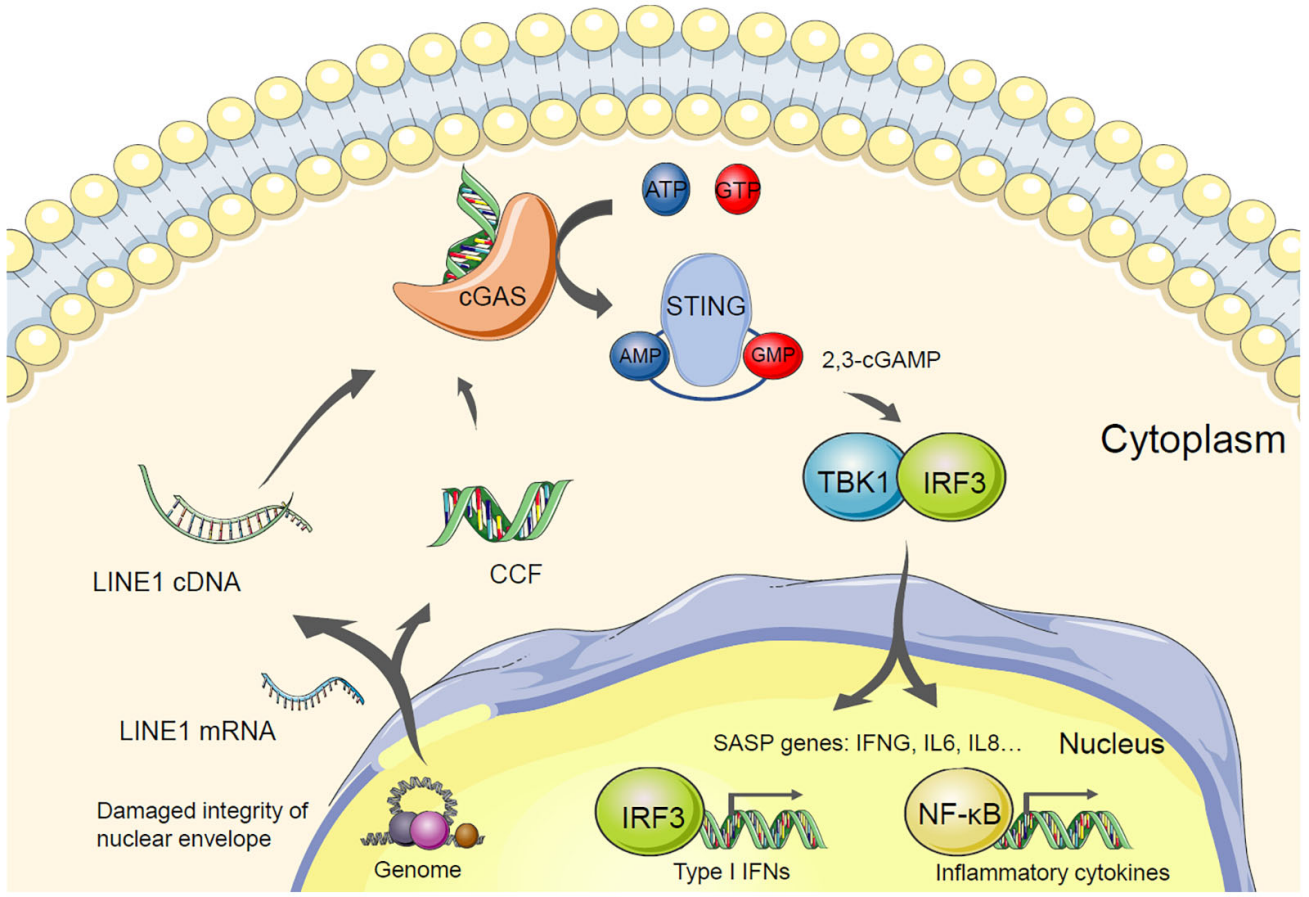
LINE1 and CCF stimulate the SASP gene expression through the cGAS‐STING innate immune pathway. When cells undergo senescence, impaired integrity of the nuclear envelope contributes to the formation of CCF. In another aspect, high levels of LINE1 mRNA were found in senescent cytosolic, which leads to increased amount of LINE1 cDNA through reverse transcription. These two sources of cytosolic DNA bind to and activate cGAS to generate the second messenger 2,3‐cGAMP. cGAMP sequentially binds to and activate STING to recruit TBK1. TBK1 facilitates the phosphorylation of IRF3 and NFkB, which function as important transcriptional factors to promote the SASP gene expression. 2,3‐cGAMP, 2,3‐cyclic GMP‐AMP; AMP, adenosine monophosphate; ATP, adenosine 5′‐triphosphate; CCF, Cytoplasmic chromatin fragment; cGAS, GMP–AMP synthase; GMP, guanosine monophosphate; GTP, guanosine 5′‐triphosphate; IRF3, interferon regulatory transcription factor3; LINE1, Long Interspersed Element 1; NFkB, nuclear factor‐kappa B; STING, stimulator of interferon genes; TBK1, tank‐binding kinase 1. [Colour figure can be viewed at wileyonlinelibrary.com]

The innate immune cGAS‐STING pathway is a critical regulator of the SASP (Fig. 3). This pathway functions to sense cytosolic DNA and induce an immune response [73]. Upon binding cytosolic DNA, cyclic GMP‐AMP Synthase (cGAS) produces 2,3‐cyclic GMP‐AMP (2,3‐cGAMP), which subsequently binds to Stimulator of Interferon Genes (STING) to trigger the activation of interferon regulatory transcription factors (IRFs), such as IRF3 [73]. Activated IRFs stimulate the transcription of inflammatory genes and therefore mediate innate immune response [73]. The cGAS‐STING signalling pathway is activated by recognizing cytoplasmic chromatin fragments (CCF), which form and accumulate during senescence with the loss of nuclear envelope integrity [74]. cGAS is similarly activated by cytoplasmic‐localized cDNA generated by the reverse transcription of de‐repressed retrotransposon LINE‐1 elements (long‐interspersed element 1) [75]. Once activated, cGAS stimulates the phosphorylation of STING and promotes NFκB transcription, which leads to the upregulation of SASP‐associated genes [76].

In OIS or RS cells, LINE‐1 acts as a driver of type I interferon (IFN‐I) signalling via cGAS‐STING activation to induce chronic inflammation [75]. In contrast, CCF‐triggered cGAS activation cannot stimulate IFN‐I in OIS or TIS human fetal diploid (HFD) cells [74]. This is likely due to p38 mitogen‐activated protein kinase (MAPK)‐mediated STING inhibition [74, 77]. However, in oxidative stress‐induced senescent mouse embryonic fibroblasts, cGAS activation triggered by CCF can induce IFN‐I production [76]. Despite their context‐dependent differences in IFN signalling activation, both CCF and LINE‐1 induce the SASP in a cGAS‐dependent manner. Given that senescence‐related inflammation is reduced in cGAS‐deficient, and in STING‐deficient, cells or mice, inhibitors of the cGAS‐STING axis have attracted broad interest [74, 78, 79]. cGAS or STING inhibitors such as G150 and H151 have been reported to have potency in the sub‐micromolar range [80, 81]. However, whether these inhibitors can be repurposed as effective senomorphics with manageable side effects remains to be determined [80, 81].

Strategies that dampen the upstream triggers of cGAS‐STING signalling might provide an equally effective therapeutic approach. For example, Lamivudine, which is a nucleotide reverse transcriptase inhibitor, has been shown to suppress LINE1 formation [75]. This correlates with a decreased inflammatory response and improved age‐associated phenotypes in naturally aged mice and in a progeroid ageing mouse model [75]. Furthermore, HDAC inhibitors impair CCF formation by damaging mitochondria‐to‐nucleus retrograde signalling, which also results in SASP suppression [82]. Given that HDAC inhibitors such as vorinostat and belinostat are already approved for the treatment of patients with haematological malignancies, it provides a promising therapeutic potential for treating age‐related diseases [83].

Overall, senomorphics aim at suppressing the SASP without interfering with the cell growth arrest of senescent cells. Despite that, senomorphics show promising efficacy in suppressing the SASP expression in multiple types of senescent cell models and animal models, strategies that target on other signalling pathways may provide additional avenues for therapeutic interventions.

## 4 Other potential strategies for treating senescence‐associated diseases

Given that senescence is accompanied by metabolic alteration, DNA damage, and stem‐like signatures, strategies targeting these alterations may remodel the senescence‐associated microenvironment for treating ageing‐related diseases [1]. Other strategies such as targeting of increased lysosomal enzyme activity or intrinsic lowered pH environment have been reviewed elsewhere [84].

### 4.1 Interventions for metabolic dysfunction

Metabolic dysfunction is known to induce senescence responses [85]. The altered metabolic status of senescent cells plays an essential role in reinforcing senescence‐associated growth arrest and in sustaining the SASP. For example, during mitochondrial dysfunction‐associated senescence (MiDAS), cytosolic NADH accumulates, resulting in a low NAD^+^/NADH ratio, which activates the energy sensor, AMPK signalling, to elicit the SASP [86]. NAD^+^ can also serve as a cofactor for sirtuin family proteins (SIRTs) and for poly‐ADP‐ribose polymerase (PARP), which both protect cells from senescence in a manner that depends on their ability to consume NAD^+^ [87, 88, 89, 90]. For example, SIRT1 and SIRT6 loss in primary human lung fibroblasts induces senescence or premature ageing with a hyperinflammatory phenotype [90, 91, 92, 93]. Sirtinol, a Sirt1 inhibitor, induces senescence‐like growth arrest and reduced MAPK signalling in human cancer cells [94]. Although NAD^+^ protects against senescence‐associated growth arrest, one study has shown that once senescence is fully established, NAD^+^ promotes inflammatory SASP during OIS and TIS [95]. Mechanistically, this is mediated by the upregulation of nicotinamide phosphoribosyl transferase (NAMPT) through the high mobility group A (HMGA) proteins [95]. An increased NAD^+^/NADH ratio suppresses the AMPK signalling pathway to enhance NFκB transcriptional activity, which upregulates the SASP [95]. Consistently, selective NAMPT inhibitors, such as FK866, suppress the SASP, inhibit tumour progression, and prevent SASP‐associated chemotherapy relapse by eliminating cancer stem‐like cells in xenograft mouse models [22]. In addition to NAD metabolism, mitochondrial deficiency, including the loss of mitochondrial proteins, the stalling of the electron transport chain, and mutations in mitochondrial DNA (mtDNA), are all known drivers of senescence [96, 97, 98]. For example, the overexpression of mitochondrial protein deacetylase SIRT3 in human fibroblasts can antagonize cellular senescence induced by high glucose [99].

Reactive oxygen species (ROS) also accumulate in mitochondria during senescence [85]. Increased ROS production activates c‐Jun N‐terminal kinase (JNK) to promote the release of CCF, which drive the SASP via the cGAS‐STING pathway [100]. Consistently, antioxidants or JNK inhibitors are sufficient to suppress CCF formation, thus reducing the SASP. Senescence is also induced in mice by the depletion of mitochondrial superoxide dismutase (SOD2), which destroys superoxide anion radicals [101]. However, ROS are vitally important signalling molecules that are involved in many cellular processes, making it a formidable challenge to inhibit them without causing deleterious effects.

### 4.2 Restoring p53 function to induce senescence

The p53/p21^cip1^ tumour suppressor pathway plays a vital role in regulating senescence [102]. p53 can be activated during senescence both in a DDR‐dependent and DDR‐independent manner. For instance, both OIS and RS are accompanied by DNA damage, which triggers the DDR cascade to phosphorylate and stabilize p53 [103, 104]. p53 can also be activated and stabilized by direct phosphorylation by p38 or by being bound by mTORC1 [105]. p53 transcriptionally stimulates p21^cip1^, which plays a critical role in mediating cell cycle exit during senescence [106, 107]. Furthermore, p53 undergoes post‐translational modifications during senescence. For example, the acetylation of lysine 320 of p53 promotes p21^cip1^ expression [108]. This contributes to the maintenance of senescence since p21^cip1^ knockout in TIS cells triggers apoptosis via caspase activation [109].

In the absence of p16, p53 inactivation prevents human senescent fibroblasts from undergoing senescence [110]. In addition, in double mutant *Tp53* ^−/−^/*Atm* ^−/−^ mice that lack both p53 and ATM, cells fail to senesce and the mice develop tumours earlier than their single knockout counterparts do [111]. These findings indicate that p53 acts as an inducer of senescence that protects cells from being cancerous. Thus, p53 might induce fast‐growing cancer cells to senescence and thus provide a potential therapeutic target for cancer treatment. Given this possibility, the activation of p53 by small molecules has been explored. For example, the ability to alter p53 activation via post‐translational modification using inauhzin and by blocking its nuclear exportation by using RG7112 has been investigated, as has restoring p53 activity via the delivery of p53 mRNA [112, 113, 114, 115, 116]. These studies also highlight the potential to combine a p53 activator with senolytics to eliminate cancer cells by first inducing their senescence, in what is known as a ‘one‐two punch’ therapeutic strategy [117].

### 4.3 Blocking senescence‐associated stemness in cancer

It is well‐known that crucial signaling components of senescence pathways, such as p16^INK4a^, p21^cip1^, and p53, are also critical regulators of cancer stem‐cell‐like phenotypes [118]. A gain of stemness in cancer cells has profound effects on tumour progression and aggressiveness [119]. For example, in a transcriptome profiling study of TIS cells, a dramatic increase in a stem‐cell transcriptional signature was observed in senescent, compared to non‐senescent, lymphomas [21]. Moreover, enhanced tumour initiation was observed in cells that had escaped from senescence through reduced level of lysine 9 trimethylated histone H3 (H3K9me3) or p53, in TIS cancer cells and in mouse models of leukaemia [21]. Strikingly, non‐stem bulk leukaemia cells gained the ability to self‐renew and could be made into leukaemia‐initiating stem cells by temporarily undergoing enforced senescence *in vitro* [21]. In another study, NAMPT inhibition significantly suppressed the senescence‐associated stemness in ovarian cancer cells that are triggered by platinum‐based chemotherapy [22]. Moreover, the combination of the NAMPT inhibitor, FK866, and cisplatin dramatically improved the survival of mice bearing ovarian tumours and was mechanistically correlated with the inhibition of stem‐cell signatures [22]. Overall, these studies provide a novel strategy for blocking senescence‐associated stemness in the treatment of human cancers.

## 5 Conclusions and perspectives

Senescence is a complicated cellular process that is characterized by a variety of phenotypic changes and pleiotropic functional consequences. These phenotypic changes are spatially and temporally dynamic and depend on stress inducers and the genetic context [120]. While the growth arrest of senescent cells is beneficial in early life, protecting healthy tissues from tumorigenesis, senescent cells are resistant to programmed cell death and stimulate chronic inflammation, thus damaging tissue homeostasis and contributing to tumorigenesis in later life. Despite the complexity of cellular senescence, emerging evidence demonstrates that senescence‐based therapeutic approaches have considerable potential for promoting healthy ageing and for combating age‐associated diseases, such as cancer [10]. Of particular therapeutic potential is the use of senolytics to eliminate senescent cells and the use of senomorphics to suppress the SASP.

Technical advancements and innovations bring new avenues for the development of novel senescence‐based interventions. The advances of whole‐genome next‐generation sequencing (NGS) are greatly accelerating biomarker identification and targeted therapy in cancer [121, 122]. The integration of big data‐mining and artificial intelligence (AI)‐based approaches holds great promise for the identification of novel biomarkers in senescence‐associated pathologies [123]. Once novel senescent biomarkers are identified, cutting‐edge therapies can be developed to treat senescence‐associated diseases [124, 125, 126]. Advances in functional genomics can also be used to dissect context‐dependent senescence‐regulating pathways, such as the use of genome‐wide CRISPR (clustered regularly interspaced short palindromic repeats)‐based screening [127, 128]. Notably, single‐cell‐based technologies, including single‐cell RNA sequencing (scRNA‐sequencing) and single‐cell split‐pool recognition of interactions by tag extension (scSPRITE) enable the 2D transcriptome and 3D epigenome to be measured in dynamic and heterogenous cell populations, such as in ageing tissues [129, 130]. Moreover, a novel method for enriching extrachromosomal circular DNA elements (eccDNAs) has been recently reported [131]. This study showed that eccDNAs can be used to activate STING in regulating the immune response in bone marrow‐derived dendritic cells [131]. However, whether eccDNA exists due to damaged chromosomal DNA and thereby stimulates cGAS‐STING signalling in the context of senescence remains to be investigated. Together, these evolving technologies will both pave the way for identifying novel targets for senescence‐based therapies and help to uncover senescence‐associated mechanisms that can be leveraged for therapeutic targeting.

## Conflict of interest

The authors declare no conflict of interest.

## Author contributions

CW, XH and RZ conceived and wrote the manuscript.

## Data Availability

Data sharing is not applicable to this review as no new data were created or analyzed in this review.
